# Response to: COVID-related upsurge in diagnoses of advanced breast cancer—is a disruption in mammography screening the one to be blamed?

**DOI:** 10.1016/j.esmoop.2021.100109

**Published:** 2021-04-10

**Authors:** A. Toss

**Affiliations:** 1 Department of Oncology and Hematology, Azienda Ospedaliero-Universitaria di Modena, Modena, Italy; 2 Department of Surgery, Medicine, Dentistry and Morphological Sciences with Transplant Surgery, Oncology and Regenerative Medicine Relevance, University of Modena and Reggio Emilia, Modena, Italy

We would like to thank the editors for giving us the opportunity to respond to the issues raised by Drs Tomasik and Braun in their letter and to clarify aspects of our study in relation to these concerns. We would also like to thank our colleagues for their interest in our paper and for taking the time to express their concerns.

In this letter to the editor, Drs Tomasik and Braun underline that the increase in late-stage breast cancer (BC) diagnosis described by our analysis might be, at least partially, explained by the significant decrease in the number of people with symptoms consulting with clinicians, rather than disruptions in mammographic screening. Particularly, this delay could be explained, first, by the adherence to public health guidelines during the pandemic, and second, by the fear of coronavirus disease itself.

Nevertheless, as shown in Table 1 of our paper,^1^ symptomatic (self-reported) BC patients did not significantly decrease from 2019 (26.9%) to the same trimester in 2020 (23.2%; *P* = 0.418). Moreover, as suggested by the colleagues, we repeated our analysis including only screen-detected nonsymptomatic cancers, and the rate of stage III BC was observed to be 0.7% (1 out of 136 patients) in 2019 and 9.6% (9 out of 94 patients) in 2020 (+8.9%). Therefore, at least in our study population, the increase in late-stage tumor diagnoses should not be attributed to delay in referrals to clinicians.

However, we agree that this is a complex matter, and it is unlikely that such relevant findings are only due to mammographic screening disruptions. Indeed, our results should be interpreted with caution and further research is needed on this topic to identify the other factors at work.

## References

[1] Toss A., Isca C., Venturelli M. (2021). Two-month stop in mammographic screening significantly impacts on breast cancer stage at diagnosis and upfront treatment in the COVID era. ESMO Open.

